# The Role of Bone Edema in Plantar Fasciitis Treated with Temperature-Controlled High-Energy Adjustable Multi-Mode Emission Laser (THEAL) and Exercise: A Prospective Randomized Clinical Trial

**DOI:** 10.3390/biomedicines12081729

**Published:** 2024-08-02

**Authors:** Ilaria Covelli, Silvana De Giorgi, Antonio Di Lorenzo, Biagio Moretti, Giuseppe Solarino, Angela Notarnicola

**Affiliations:** 1 Orthopedics Unit, Department of Translational Biomedicine and Neuroscience “DiBraiN”, School of Medicine and Surgery, University of Bari, General Hospital, Piazza Giulio Cesare 11, 70124 Bari, Italy; ilariacovelli@yahoo.it (I.C.); silvana.degiorgi@uniba.it (S.D.G.); biagio.moretti@uniba.it (B.M.); giuseppe.solarino@uniba.it (G.S.); 2 Interdisciplinary Department of Medicine, University of Study of Bari, General Hospital, Piazza Giulio Cesare 11, 70124 Bari, Italy; antoniodilorenzo95@gmail.com

**Keywords:** high-intensity laser therapy, exercise, plantar fasciitis, bone edema

## Abstract

Plantar fasciitis is one of the most common causes of foot pain; in 35% of cases, it is also associated with bone edema of the heel. The aim of this study was to investigate the relationship between bone edema and the outcomes of temperature-controlled high-energy adjustable multi-mode emission laser (THEAL) and/or exercises in patients with plantar fasciitis. A prospective randomized clinical trial was designed, in which 48 patients suffering from plantar fasciitis, with or without bone edema, were treated with temperature-controlled high-energy adjustable multi-mode emission laser and exercises (the laser group) or with exercises only (the control group). The patients were evaluated at recruitment (T0) and at 2 (T1) and 6 months (T2), monitoring pain (with the Visual Analogue Scale), functionality (with the Foot Function Index), perception of improvement (with the Roles and Maudsley Score), and fascia thickness (with ultrasound examination). In both groups, there was a significant improvement in pain, functional recovery, perception of remission, and a reduction in plantar fascia thickness at T1 and T2. The laser group presented statistically better values at T2 for the Roles and Maudsley Score (z: 2.21; 0.027). The regression analysis showed that a greater reduction in fascia thickness occurred in the laser group (*p*-value: 0.047). In conclusion, the two conservative treatments were effective in patients suffering from plantar fasciitis, even in the presence of bone edema, but with lesser results.

## 1. Introduction

Plantar fasciitis is a very common disease in the orthopedic field. Heel pain is generally more acute in the morning and decreases during the day with movement [1]. It is characterized by inflammation and the simultaneous degeneration of the insertion of the fascia that covers the muscles present at the sole of the foot, with progressive evolution with calcification at the insertion. The incidence of plantar fasciitis is between 9 and 20% of the population, with a higher presence in middle-aged obese women and young male runners [2]. X-rays allow us to check for deformities or the presence of a spur under the heel, while ultrasound studies the integrity of the connective tissue and its thickening. Magnetic resonance imaging (MRI) evaluates the heel and plantar fascia and checks for stress fractures, tarsal tunnel syndrome, and Achilles tendinopathy [3]. Bone edema can be associated with plantar fasciitis. This nonspecific sign could generally be the result of avulsive trauma, stress fractures, or intrabony fractures. These findings are similar to those found in epicondylitis, in which repeated overuse could be the cause of increased bone edema [4].

In the literature, bone edema at the heel is present on MRI in 35% of patients suffering from plantar fasciitis [5,6]. In shock wave treatment, the presence of bone edema of the calcaneus could be a highly predictive factor for a better response [7]. The presence of fascia thickening and soft tissue signal changes does not affect the clinical response, whereas the presence of bone edema may result in a better response to biostimulation.

Stretching exercises of the posterior muscle chain and plantar fascia, anti-inflammatories, cortisone infiltration, and biostimulation with physical therapies (laser therapy, shock waves, ultrasound therapy, etc.) are the standard treatments for plantar fasciitis [8]. In forms that do not respond to conservative treatments, surgical options are used.

In laser therapy, there is an emission of electromagnetic radiation, characterized by monochromaticity, collimation, coherence, and the high brightness of the light [9]. These characteristics guarantee biological effects in cells: increased ATP production, increased activity of membrane enzymes, increased DNA and RNA synthesis, and the acceleration of electrolyte exchange between the cell and the extra-cellular space [9]. Furthermore, at the tissue level, vasodilation, reduction in intra-capillary pressure, increased excitability of nerve fibers, and the stimulation of the immune response occur [10]. Recently, the application of high-intensity laser therapy has been finding space in the treatment of tendinopathies supported by the proliferative, analgesic, and inflammation-modulating effects on the musculoskeletal system [11,12,13]. 

The aim of this study was to investigate the relationship between bone edema and the outcomes of temperature-controlled high-energy adjustable multi-mode emission laser and/or exercises in patients with plantar fasciitis.

## 2. Materials and Methods

We conducted a prospective randomized clinical trial. Patients who came to our clinic with a diagnosis of plantar fasciitis, with or without bone edema, were included in our study and gave written informed consent to participate. This study was conducted in accordance with the Declaration of Helsinki and approved by the Territorial Ethics Committee of the University Hospital Consortium of the Polyclinic of Bari (protocol code 7741, 17 May 2023). The study is registered at ClinicalTrials.gov (NCT05925777).

The inclusion criteria are as follows: history of heel pain for at least 3 months before enrollment; tenderness on palpation of the medial calcaneal tubercle or proximal plantar fascia; and plantar fascia thickness of 4.0 mm or greater [14,15,16].

The exclusion criteria were very strict: age under 18 years; an anamnestic history of systemic diseases; pregnancy; previous lower limb surgery; a diagnosis of fibromyalgia, neurologic disease, Achilles tendinopathy, acute ankle sprain, tarsal tunnel syndrome, or heel spur syndrome; a body mass index (BMI) greater than 35 kg/m^2^; wounds or infections in the area; altered sensation in the area to be treated; changes in skin pigmentation in the area to be treated; metal implants in the treatment area; treatment with corticosteroids within the last six weeks; diagnosis of neurological heel pain; diagnosis of systemic inflammatory arthritis; other acute pathologies requiring treatment; other painful conditions requiring painkillers; and a tumor, cardiac pacemaker, or other device [17,18].

For each patient, the epidemiological and anthropometric variables (age, gender, weight, height, BMI, smoking habit) and the clinical characteristics (site of the pathology, heel spur, duration, previous therapies, and clinical outcomes) were set. Each patient was evaluated at the beginning of the treatment (T0) and after two (T1) and six months (T2). At the time of the clinical evaluation, all patients received indications on education and the prevention of overload and footwear advice.

Heel pain at the beginning of walking was assessed by asking the patient to express the intensity of the pain on a Visual Analogue Scale from 0 to 10 cm (“0” = no pain and “10” = the most intense pain) [19]. Each patient expressed the impact of the foot pathology on functionality in terms of pain, disability, and functional limitation by self-administering the Foot Functional Index (FFI), whose score ranges from a disability of 0% to 100% [20]. At T1 and T2, the Roles and Maudsley (RM) Score was used to quantify the perception of improvement by the patient (ranging from 1 for excellent to 4 for poor) [21].

On enrollment, an echography of the plantar fascia and an MRI of the foot were evaluated. The ultrasound measurement was performed with the patient in the prone position and the foot protruding from the table. Acoustic gel was placed at the heel and the ultrasound probe was subsequently placed perpendicular to the insertion of the plantar fascia on the heel. The thickness of the plantar fascia was measured at its proximal end near its insertion into the calcaneus [17,22]. An MRI evaluation of the entire foot was performed and for the calcaneal bone edema, the diagnosis of bone edema was formulated for a poorly defined area with high-signal intensity on STIR images and low-signal intensity on T1-weighted SE images [23,24]. 

At two and six months, the patients were re-evaluated using an ultrasound to monitor the thickness and appearance of the plantar fascia. In the case of the first finding of bone edema, the MRI was also repeated at the two subsequent follow-ups to monitor the bone edema. Any adverse effects were recorded: increased pain, petechiae, ecchymosis, and vagal crisis.

Patients were randomized into one of two groups:

- The control group: treatment with stretching exercises.
- The laser group: THEAL treatment (temperature-controlled high-energy adjustable multi-mode emission laser) and stretching exercises. The two treatments began together; on the same day, each patient first carried out the laser session and then the exercises.

In each group, an equal number of patients with and without osseous edema were enrolled in order to avoid possible interference caused by the presence/absence of this condition.

### 2.1. Control Group

Patients were instructed to perform four daily stretching exercises for 6 weeks [15]. Each stretching exercise was maintained for 30 s and repeated 3 times a day. At recruitment, patients were instructed on how to perform the exercises. The first stretching treatment session was conducted by an expert physiotherapist in order to demonstrate the exercise program. Once a week, the method and frequency of carrying out the exercises were checked.

**Figure 1:**
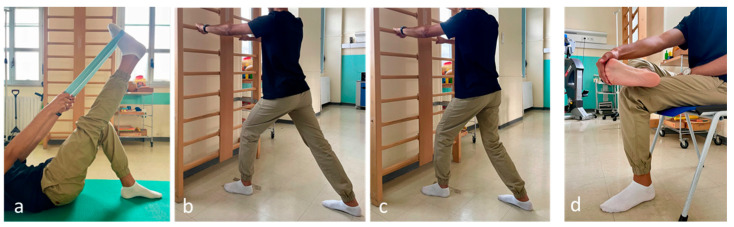
Exercises: (**a**) straight leg raise in supine position; (**b**) plantar flexor muscles stretch with knee extended; (**c**) plantar flexor muscles stretch with knee flexed; (**d**) plantar fascia stretch.

1. Stretching the hamstrings and ankle plantar flexors (supine straight leg raise).
2. Self-stretching of the leg muscles: the patient bends forward in a standing position with the affected foot furthest from the wall, keeping the heel on the floor.
3. The soleus muscle is stretched with the knee flexed and the gastrocnemius muscle with the knee extended.
4. Self-stretching of the plantar fascia: in a sitting position, the patient crosses the affected foot over the contralateral thigh and performs a passive extension of the metatarsophalangeal joints (Figure 1).

### 2.2. Laser Group

The temperature-controlled high-energy adjustable multi-mode emission laser (THEAL) was delivered with an Ixyon XP device (Mectronic, Bergamo, Italy) which allows for the delivery of 4 wavelengths (650 nm, 810 nm, 980 nm, and 1064 nm), with continuous and pulsed mode and an average power of up to 30 W, administering 10 sessions every other day. Each single session was divided into three phases, the energy parameters of which are described in Table 1. The protocol was defined in accordance with the literature [11].

In the first phase, the activation of the inguinal, popliteal, and malleolar lymphatic stations was envisaged for 1 min 40 s for each station, accompanied by very light manual mechanical pressure (1800 J). The applicator used was a small IR (Infrared), and 600 J was delivered, with a thermal control ranging from 40 to 43 °C.

The second phase involved the treatment of the plantar fascia, with anti-inflammatory and biostimulant action, identifying the treatment area by approximating the surface to a 5 cm × 5 cm square and inserting the data into the device software in order to determine the time of the therapy (4 min 18 s) (1750 J). The applicator was a large IR, delivering a dose of 70 J/cm^2^ with a variable thermal control of 38–42 °C.

The third phase aimed to treat the latent trigger points (TPs) of the tibialis and triceps surae muscles, with analgesic and decontracting action (120 J). The applicator used was IR-collimated (20 s), delivering 60 J × TP, with thermal control stopping at 42 °C.

The patients performed the stretching exercises at the same time, as expected in the control group.

### 2.3. Sample Size Calculation

Sample estimation was performed using the t-test, and a significance level (alpha) of 0.05 and a test power of 80% were set. A sample size of 44 subjects was estimated; assuming a 10% loss to follow-up, the number of subjects to be recruited was 48 (24 per group). This effect was selected as the smallest effect that would be important to detect, meaning that any smaller effect would have no clinical or substantive significance.

The assignment to the groups was carried out by randomization, with the two groups being homogeneous for the covariates, sex, and diagnosis of bone edema (in relation to the hypothesis that the presence of bone edema could be a prognostic factor influencing the response). The two groups were designed to contain the same number of subjects with and without osseous edema.

Both the sample size assessment and the randomization were carried out via STATA MP17^®^ software (IBM^®^, Armonk, NY, USA). 

### 2.4. Statistical Analysis

The primary endpoint was pain reduction, assessed with the VAS scale. The secondary endpoint was clinical improvement, assessed with FFI and the Roles and Maudsley score. The tertiary endpoint was an instrumental improvement, which was assessed through an evaluation of the reduction in the thickness of the plantar fascia (assessed by ultrasound) and the reduction in bone edema (assessed by MRI), when present at T0.

The data were collected on paper forms and then computerized into an Excel database. To estimate the sample size, a VAS value at enrollment (T0) of 6.5 was assumed for both groups [17,25], with a mean reduction at the primary endpoint (T2) of 4.1 in the control group [25] and 2.6 in the laser group, and with a standard deviation for both groups of 1.7.

Categorical variables were described as percentages and proportions. Quantitative variables were described as means (±standard deviations). The changes in the VAS and FFI values and the echography-measured thickness of the plantar fascia between the study and control group over 6 months were studied via Student’s *t*-test, to verify whether or not the use of laser therapy had improved the outcome of the treatment. The Roles and Maudsley scores at T1 and T2 were also compared between the two groups via Student’s *t*-test for repeated measures; however, the T2 scores were compared via the Mann‒Whitney test due to the non-normality of the variable’s distribution. The presence of RMI improvement between T0 and T2 was studied via the Chi-squared test.

Multivariable regression was employed for all considered endpoints. The outcomes were represented by the change over time in the VAS and FFI, by the echography improvement, and by the RM score at T2. The main determinants that were studied were the use of laser therapy and the presence of bone edema, while sex, age, smoking habit, body mass index (BMI), laterality, months since diagnosis, cardiovascular comorbidities, metabolic comorbidities, previous non-steroid anti-inflammatory therapy, previous therapy, and the presence of spurs were taken into consideration as confounders. The association was expressed as an adjusted odds ratio (aOR) and a 95% confidence interval (95% CI).

A *p*-value < 0.05 was chosen as an indicator of statistical significance. The database was built via Microsoft Excel^®^ 2019. All calculations were performed via STATA MP17^®^ software.

## 3. Results

### 3.1. Population Characteristics

**Figure 2:**
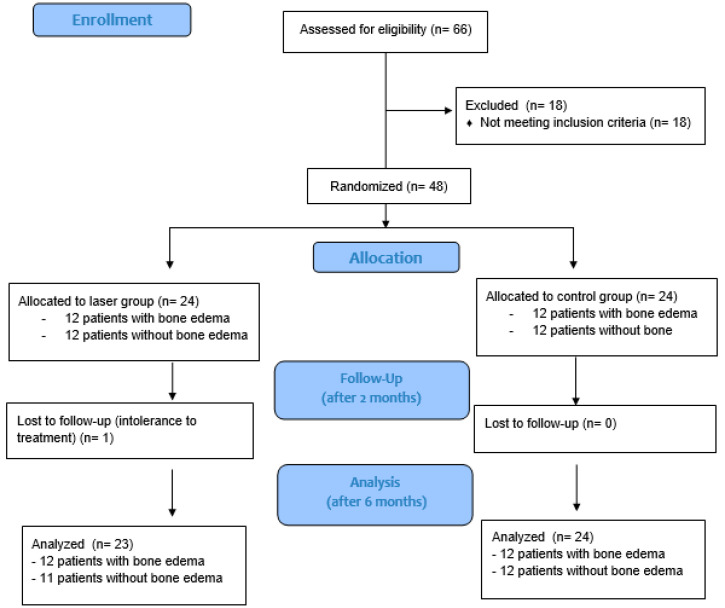
Flow chart showing patient recruitment.

The study enrolled 48 subjects, equally distributed between the laser group and the control group. A patient from the laser group asked to drop out of the study due to significant pain making it impossible to continue the current therapy (final distribution: 23 subjects in the laser group and 24 in the control group) (Figure 2). The population’s characteristics are summarized in Table 2. The mean age was 58.04 years (±9.13 years), while the mean BMI was 26.16 kg/m^2^ (±3.07 kg/m^2^). The mean time since diagnosis was 13.14 months (±13.42 months).

### 3.2. Endpoints

A significant reduction in the mean VAS from T0 to T1 was observed in both the laser group (t: 8.25; *p*-value < 0.001) and the control group (t: 9.01; *p*-value < 0.001). The mean reduction was −3.28 (±1.88). This significance was confirmed at T2 for both groups (laser group: t: 9.81; *p*-value < 0.001; control group: t: 8.90; *p*-value < 0.001), and the mean variation from T0 to T2 was −4.34 (±2.24).

A significant reduction from T0 to T1 was also observed for the FFI of both the laser group (t: 5.98; *p*-value < 0.001) and the control group (t: 5.80; *p*-value < 0.001). The mean reduction was −22.21 (±18.11). This significant reduction was confirmed from T0 to T2 in both the laser group (t: 7.73; *p*-value < 0.001) and the control group (t: 7.34; *p*-value < 0.001), with a mean reduction of −30.05 (±19.15).

**Figure 3:**
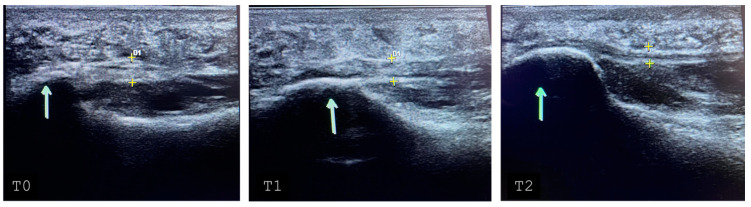
Ultrasound of the plantar fascia of a patient suffering from plantar fasciitis belonging to the laser group. At T0, there was a band thickness of 5 mm, which reduced to 4 mm at T1 and T2 to 3 mm. The arrows point to the heel; + signs indicate plantar fascia thickness; the D1 (1st distance) indicates measurement.

**Figure 4:**
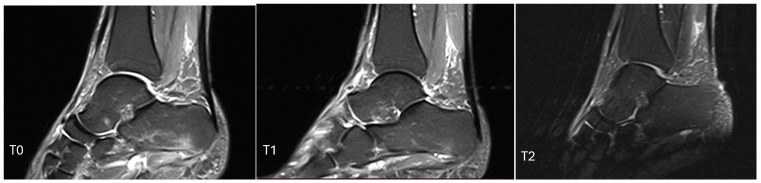
MRI of the calcaneus in a patient affected by plantar fasciitis in the laser group. At T0, the presence of bone marrow edema of the calcaneus was detected, which resolved at T1 and T2.

The mean RM score at T1 was 1.96 (±0.88), while it dropped to 1.68 (±0.89) at T2. When measured via echography, the fascia plantaris of the enrolled subjects had a mean thickness reduction of −0.57 mm (±0.74 mm) between T0 and T2; this reduction was significant for both the laser group (t: 4.67; *p*-value < 0.001) and the control group (t: 2.85; *p*-value: 0.005) (Figure 3). Finally, 12.77% of the patients (6/47) showed MRI bone edema remission over time (Figure 4). No adverse effects were recorded in the two groups. The VAS, FFI, and RM score changes over time are described in Table 3.

### 3.3. Inferential Statistics

The laser group showed no significant improvement in terms of either the MRI (Chi2: 0.86; *p*-value: 0.352) or the echography evaluation (t: 1.47; *p*-value: 0.149). The VAS modification over time was also not significantly different between the laser and control groups (t: 0.67; *p*-value: 0.506). The FFI change over time was not significantly different either (t: 0.07; *p*-value: 0.942).

The two groups did not show a significantly different RM score at T1 (t: 0.99; *p*-value: 0.324). However, a significant improvement was observed in the RM score at T2 (z: 2.21; 0.027) [Mann‒Whitney test due to non-normal distribution].

### 3.4. Regression Analysis

The VAS variation over time proved to be significantly impacted by the presence of bone edema; in particular, subjects with edema showed a significantly lower reduction (aOR: 1.60; 95% CI: 0.03–3.18; *p*-value: 0.047). Similarly, the FFI reduction was significantly lower in the case of bone edema (aOR: 14.43; 95% CI: 0.81–28.05; *p*-value: 0.039). All other tested independent variables were not significant in determining the changes in the VAS and FFI over time (*p*-value > 0.05). 

The echography improvement, on the contrary, was significantly influenced by laser therapy, with exposed subjects having a greater reduction in the thickness of the fascia plantaris (aOR: −0.45; 95% CI: −0.89–−0.01; *p*-value: 0.047). Previous physiotherapy also showed a significant influence, with reduced improvements over time (aOR: 0.62; 95% CI: 0.58–1.17; *p*-value: 0.032).

Finally, the RM score at T2 was significantly lower in males (aOR: −0.81; 95% CI: −1.39–−0.23; *p*-value: 0.008) and in younger subjects (aOR: 0.36; 95% CI: 0.01–0.07; *p*-value: 0.022).

## 4. Discussion

In this randomized study, we compared two different conservative therapy methods in plantar fasciitis treatment. To the best of our knowledge, this is the first study that evaluates the high-intensity laser therapy in fasciitis plantar also in the contextual presence of calcaneal bone edema. 

We found that the two protocols (THEAL and exercises or exercises only) resulted in a significant improvement in pain, functional recovery, and the perception of remission at T1 and T2. These improvements were also found in the presence of bone edema, although to a lesser extent than with the VAS and FFI. The combined treatment of temperature-controlled high-energy adjustable multi-mode emission laser and exercises had a more important impact on the reduction in thickness of the plantar fascia and on the perception of improvement at T2. The improvement in Roles and Maudsley score was more significant in men and younger subjects.

These results are in agreement with previous studies that investigated high-intensity laser treatment (with a wavelength of 1064 nm) in plantar fasciitis. Ordahan et al. [26], for instance, demonstrated the effectiveness of high-intensity laser therapy vs. low-intensity laser therapy, in association with stretching and the use of an insole. Yesil et al. [27] verified that high-intensity laser therapy, in association with therapeutic exercise, has resulted in improvements in dynamic baropodometric measurements compared to placebo. On the other hand, other authors, such as Tkocz and colleagues [18] and Naruseviciute and Kubilius [17], did not find significant differences in favor of high-intensity laser in the treatment of plantar fasciitis.

In plantar fasciitis, histological analysis has demonstrated degenerative changes at the level of the enthesis with a deterioration in collagen fibers, increased secretion of connective proteins, the focal area of fibroblast proliferation, and increased vascularization [28,29]. In the literature, it is reported that calcaneal bone edema is found in 35% to 79% of patients suffering from plantar fasciitis clinically characterized by nocturnal pain [7,16,29,30]. Therefore, some authors suggest modifying the definition of this pathology with the term “plantar fasciosis” [29].

Various etiopathogenetic hypotheses have been put forward: One is that the inflammatory process affecting the plantar fascia is responsible for local vascular ectasia, with a consequent increase in water in the skeletal compartment and a subsequent increase in intraosseous pressure [29]. Another hypothesis is that it is associated with fatigue or a stress injury to the bone, as a consequence of the pain and dysfunction of the heel [31]. 

Patients in both groups in this study performed fascia and hamstring stretching exercises. A systemic review confirms the effectiveness of stretching in reducing symptoms of plantar fasciitis [32]. The plantar fascia is made up of type 1 collagen fibers [32], the synthesis of which is stimulated by stretching [33,34,35], resulting in clinical and functional improvements. 

In the laser group, a high-intensity laser cycle was administered, with the simultaneous emission of four different wavelengths: 650 nm, which determines proliferative effects of mesenchymal cells, collagen, and fibronectin [35,36,37]; 810 nm, which accelerates ATP production and promotes tendon regeneration [37]; 980 nm, which stimulates thermal and mechanical receptors and induces analgesic effects through gate control [38]; and 1064 nm, which induces anti-inflammatory effects on connective tissue [39]. The benefits found in reducing the thickness of the plantar fascia in these patients can be traced back to these tissue-remodeling stimuli.

Clinical and functional improvements were also found in the presence of bone edema, although to a lesser extent than in patients in whom bone edema was absent. In clinical practice, laser therapy currently has no indications in the treatment of bone tissue pathologies, despite encouraging results from experimental studies, in which laser therapy stimulated the proliferation of osteoblasts [40], induced neo-angiogenesis [41,42], and determined an anti-edema effect on bone tissue [43]. On the other hand, the biological stimulus of the high-intensity laser has proven to be able to significantly reduce the thickness of the fascia, confirming the biostimulating effects on the connective tissue [44]. Limitations of the present study include the lack of a control group receiving no treatment and the number of patients, which could be expanded in subsequent studies. A third limitation of this study is that, despite periodic checks, it was not possible to be certain how often or for how long the patients in the two groups performed their exercises. The absence of a placebo group is another weakness of this study, which could have brought a greater impact on the conclusions.

Furthermore, due to the lack of longer-term follow-ups, it was not possible to verify the persistence of the benefits beyond 6 months. On the other hand, this study made it possible to monitor the effects of high-intensity laser therapy even in the presence of bone edema, confirming that this may be responsible for a lower response to conservative treatment. Subsequent studies could investigate the effects of other therapies, such as extracorporeal shock wave therapy or pulsed electromagnetic fields in this pathological model.

## 5. Conclusions

In conclusion, the two investigated treatments of temperature-controlled high-energy adjustable multi-mode emission laser and exercises or exercises alone were effective in the treatment of plantar fasciitis, even in the presence of bone edema. On the other hand, when calcaneal edema was present, the clinical and functional response was lower. The high-intensity laser treatment resulted in a significant reduction in the thickness of the plantar fascia, in accordance with the biostimulation effects of the connective tissue, and a better perception of clinical improvement at the end of the study.

## Figures and Tables

**Table 1:** Energetic parameters of the three phases of each session of the THEAL treatment. The first phase aims to activate the lymph node stations activation, the second phase, the treatment of the plantar fascia, and the third phase, the action on the trigger points. CW: continuous-wave; PBM: photobiomodulation mechanism; E^2^C: superpulsed emission.

Phase	Wavelength	Power	Modality of Emission	Source	Total Energy	Thermal Control
1st						
	650 nm	1 W	CW	Small Infra-Red	1800 J	40–43 °C
	810 nm	2.5 W	CW			
	980 nm	1.2 W	CW			
	1064 nm	1.2 W	CW			
2nd						
	650 nm	2 W	CW	Large Infra-Red	1750 J	38–42 °C
	810 nm	10 W	PBM			
	980 nm	1 W	E^2^C			
	1064 nm	1 W	E^2^C			
3rd						
	650 nm	2 W	CW	Collimated Infra-Red	120 J	42 °C
	810 nm	2 W	CW			
	980 nm	1 W	CW			
	1064 nm	1 W	CW			
Total	-	-	-	-	3670 J	-

**Table 2:** Population characteristics.

Characteristics		n	%
Sex	Male	24	51.06
	Female	23	48.94
Smoking habit	No	42	89.36
	Yes	5	10.64
Cardiovascular comorbidities	No	30	63.83
	Yes	17	36.17
Metabolic comorbidities	No	31	65.96
	Yes	16	34.04
Previous non-steroid anti-inflammatory therapy	No	34	72.34
	Yes	13	27.66
Previous physiotherapy	No	31	65.96
	Yes	16	34.04
Presence of bone edema	No	23	48.94
	Yes	24	51.06
Presence of spurs	No	19	40.43
	Yes	28	59.57
Laterality	Right foot	17	36.17
	Left foot	30	63.83

**Table 3:** Description of the main endpoints over time; each endpoint is described as mean ± standard deviation.

	VAS			FFI			RM	
	T0	T1	T2	T0	T1	T2	T1	T2
Laser group	7.35 ± 1.55	3.70 ± 2.20	2.78 ± 1.95	47.86 ± 17.22	25.83 ± 17.21	17.59 ± 15.44	1.83 ± 0.83	1.39 ± 0.66
Control group	7.33 ± 1.52	4.42 ± 1.77	3.21 ± 2.02	50.07 ± 16.74	27.69 ± 19.50	20.22 ± 19.21	2.08 ± 0.93	1.96 ± 1.00
Overall	7.34 ± 1.52	4.06 ± 2.00	3.00 ± 1.98	48.98 ± 16.83	26.78 ± 18.24	18.93 ± 17.33	1.96 ± 0.88	1.68 ± 0.89

## Data Availability

The datasets generated and/or analyzed during the current study are available from the corresponding author upon reasonable request due to privacy.

## References

[1] Klein S.E., Dale A.M., Hayes M.H., Johnson J.E., McCormick J.J., Racette B.A. (2012). Clinical presentation and self-reported patterns of pain and function in patients with plantar heel pain. Foot Ankle Int..

[2] Hill C.L., Gill T.K., Menz H.B., Taylor A.W. (2008). Prevalence and correlates of foot pain in a population-based study: The North West Adelaide health study. J. Foot Ankle Res..

[3] Grasel R.P., Schweitzer M.E., Kovalovich A.M., Karasick D., Wapner K., Hecht P., Wander D. (1999). MR imaging of plantar fasciitis: Edema, tears, and occult marrow abnormalities correlated with outcome. AJR Am. J. Roentgenol..

[4] Herber S., Kalden P., Kreitner K.F., Riedel C., Rompe J.D., Thelen M. (2001). MRI in chronic epicondylitis humeri radialis using 1.0 T equipment--contrast medium administration necessary?. Rofo.

[5] Berkowitz J.F., Kier R., Rudicel S. (1991). Plantar fasciitis: MR imaging. Radiology.

[6] Sutera R., Iovane A., Sorrentino F., Candela F., Mularo V., La Tona G., Midiri M. (2010). Plantar fascia evaluation with a dedicated magnetic resonance scanner in weight-bearing position: Our experience in patients with plantar fasciitis and in healthy volunteers. Radiol. Med..

[7] Maier M., Steinborn M., Schmitz C., Stäbler A., Köhler S., Pfahler M., Dürr H.R., Refior H.J. (2000). Extracorporeal Shock Wave Application for Chronic Plantar Fasciitis Associated with Heel Spurs: Prediction of Outcome by Magnetic Resonance Imaging. J. Rheumatol..

[8] Rhim H.C., Kwon J., Park J., Borg-Stein J., Tenforde A.S. (2021). A Systematic Review of Systematic Reviews on the Epidemiology, Evaluation, and Treatment of Plantar Fasciitis. Life.

[9] Tafur J., Mills P.J. (2008). Low-intensity light therapy: Exploring the role of redox mechanisms. Photomed. Laser Surg..

[10] Prindeze N.J., Moffatt L.T., Shupp J.W. (2012). Mechanisms of action for light therapy: A review of molecular interactions. Exp. Biol. Med..

[11] Notarnicola A., Covelli I., De Giorgi S., Moretti B. (2024). High intensity laser therapy in the treatment of tendinopathy: A brief narrative review and update of current literature. Minerva Orthop..

[12] Notarnicola A., Maccagnano G., Rifino F., Pesce V., Gallone M.F., Covelli I., Moretti B. (2017). Short-term effect of shockwave therapy, temperature controlled high energy adjustable multi-mode emission laser or stretching in Dupuytren’s disease: A prospective randomized clinical trial. J. Biol. Regul. Homeost. Agents.

[13] Notarnicola A., Maccagnano G., Tafuri S., Forcignanò M.I., Panella A., Moretti B. (2014). CHELT therapy in the treatment of chronic insertional Achilles tendinopathy. Lasers Med. Sci..

[14] McPoil T.G., Martin R.L., Cornwall M.W., Wukich D.K., Irrgang J.J., Godges J.J. (2008). Heel pain–plantar fasciitis: Clinical practice guildelines linked to the international classification of function, disability, and health from the orthopaedic section of the American Physical Therapy Association. J. Orthop. Sports Phys. Ther..

[15] Renan-Ordine R., Alburquerque-Sendín F., de Souza D.P., Cleland J.A., Fernández-de-Las-Peñas C. (2011). Effectiveness of myofascial trigger point manual therapy combined with a self-stretching protocol for the management of plantar heel pain: A randomized controlled trial. J. Orthop. Sports Phys. Ther..

[16] McMillan A.M., Landorf K.B., Cotchett M.P., Menz H.B., Gregg J.M., De Luca J. (2013). Hyperemia in plantar fasciitis determined by power doppler ultrasound. J. Orthop. Sports Phys. Ther.

[17] Naruseviciute D., Kubilius R. (2020). The effect of high-intensity versus low-level laser therapy in the management of plantar fasciitis: Randomized participant blind controlled trial. Clin. Rehabil..

[18] Tkocz P., Matusz T., Kosowski Ł., Walewicz K., Argier Ł., Kuszewski M., Hagner-Derengowska M., Ptaszkowski K., Dymarek R., Taradaj J. (2021). A Randomised-Controlled Clinical Study Examining the Effect of High-Intensity Laser Therapy (HILT) on the Management of Painful Calcaneal Spur with Plantar Fasciitis. J. Clin. Med..

[19] Reed M.D., Van Nostran W. (2014). Assessing pain intensity with the visual analog scale: A plea for uniformity. J. Clin. Pharmacol..

[20] Budiman-Mak E., Conrad K.J., Roach K.E. (1991). The Foot Function Index: A measure of foot pain and disability. J. Clin. Epidemiol..

[21] Roles N.C., Maudsley R.H. (1972). Radial tunnel syndrome: Resistant tennis elbow as a nerve entrapment. J. Bone Jt. Surg. Br..

[22] Karabay N., Toros T., Hure C. (2007). Ultrasonographic evaluation in plantar fasciitis Comparative Study. J. Foot Ankle Surg..

[23] Ridge S.T., Johnson A.W., Mitchell U.H., Hunter I., Robinson E., Rich B.S., Brown S.D. (2013). Foot bone marrow edema after a 10-wk transition to minimalist running shoes. Med. Sci. Sports Exerc..

[24] Fernandez-Canton G., Casado O., Capelastegui A., Astigarraga E., Larena J.A., Merino A. (2003). Bone marrow edema syndrome of the foot: One year follow-up with MR imaging. Skeletal Radiol..

[25] Kamonseki D.H., Gonçalves G.A., Yi L.C., Lombardi I.J. (2016). Effect of stretching with and without muscle strengthening exercises for the foot and hip in patients with plantar fasciitis: A randomized controlled single-blind clinical trial. Man. Ther..

[26] Ordahan B., Karahan A.Y., Kaydok E. (2018). The effect of high-intensity versus low-level laser therapy in the management of plantar fasciitis: A randomized clinical trial. Lasers Med. Sci..

[27] Yesil H., Dundar U., Toktas H., Eyvaz N., Yeşil M. (2020). The effect of high intensity laser therapy in the management of painful calcaneal spur: A double blind, placebo-controlled study. Lasers Med. Sci..

[28] Jarde O., Diebold P., Havet E., Boulu G., Vernois J. (2003). Degenerative lesions of the plantar fascia: Surgical treatment by fasciectomy and excision of the heel spur. A report on 38 cases. Acta Orthop. Belg..

[29] Lemont H., Ammirati K.M., Usen N. (2003). Plantar fasciitis: A degenerative process (fasciosis) without inflammation. J. Am. Podiatr. Med. Assoc..

[30] Drake C., Whittaker G.A., Kaminski M.R., Chen J., Keenan A.M., Rathleff M.S., Robinson P., Landorf K.B. (2022). Medical imaging for plantar heel pain: A systematic review and meta-analysis. J. Foot Ankle Res..

[31] Cetin A., Kiratli P., Ceylan E., Sivri A., Dincer F. (2001). Evaluation of chronic plantar fasciitis by scintigraphy and relation to clinical parameters. J. Musculoskelet. Pain.

[32] Sweeting D., Parish B., Hooper L., Chester R. (2011). The effectiveness of manual stretching in the treatment of plantar heel pain: A systematic review. J. Foot Ankle Res..

[33] Stecco C., Corradin M., Macchi V., Morra A., Porzionato A., Biz C., De Caro R. (2013). Plantar fascia anatomy and its relationship with Achilles tendon and paratenon. J. Anat..

[34] Langberg H., Ellingsgaard H., Madsen T., Jansson J., Magnusson S.P., Aagaard P., Kjaer M. (2007). Eccentric rehabilitation exercise increases peritendinous type I collagen synthesis in humans with Achilles tendinosis. Scand. J. Med. Sci. Sports.

[35] Notarnicola A., Maccagnano G., Tafuri S., Gallone M.F., Moretti L., Moretti B. (2017). High level laser therapy for the treatment of lower back pain: Clinical efficacy and comparison of different wavelengths. J. Biol. Regul. Homeost. Agents.

[36] Hamilton H.K., Dover J.S., Arndt K.A. (2014). Successful treatment of disfiguring hemosiderin-containing hyperpigmentation with the Q-switched 650-nm wavelength laser. JAMA Dermatol..

[37] Lopes-Martins R.A., Albertini R., Martins P.S., Bjordal J.M., FariaNeto H.C. (2005). Spontaneous effects of low-level laser therapy (650nm) in acute inflammatory mouse pleurisy induced by Carrageenan. Photomed. Laser Surg..

[38] Byrnes K.R., Waynant R.W., Ilev I.K., Wu X., Barna L., Smith K., Heckert R., Gerst H., Anders J.J. (2005). Light promotes regeneration and functional recovery and alters the immune response after spinal cord injury. Lasers Surg. Med..

[39] Anderson P.R. (1999). Cutaneous Laser Surgery.

[40] Coombe A.R., Ho C.T., Darendeliler M.A., Hunter N., Philips J.R., Chapple C.C., Yum L.W. (2001). The effects of low level laser irradiation on osteoblastic cells. Clin. Orthod. Res..

[41] Ribeiro D.A., Matsumoto M.A. (2008). Low-level laser therapy improves bone repair in rats treated with anti-inflammatory drugs. J. Oral Rehabil..

[42] Shibata M., Kodani I., Osaki M., Araki K., Adachi H., Ryoke K., Ito H. (2005). Cyclo-oxygenase-1 and -2 expression in human oral mucosa, dysplasias and squamous cell carcinomas and their pathological significance. Oral Oncol..

[43] Baek W.Y., Byun I.H., Yun I.S., Kim J.Y., Roh T.S., Lew D.H., Kim Y.S. (2017). The effect of light-emitting diode (590/830 nm)-based low-level laser therapy on posttraumatic edema of facial bone fracture patients. J. Craniomaxillofac. Surg..

[44] Notarnicola A., Covelli I., Macchiarola D., Bianchi F.P., Cassano G.D., Moretti B. (2023). The Efficacy of Temperature-Controlled High-Energy Polymodal Laser Therapy in Tendinopathy of the Shoulder. J. Clin. Med..

